# Polychromatic Light Exposure as a Therapeutic in the Treatment and Management of Parkinson's Disease: A Controlled Exploratory Trial

**DOI:** 10.3389/fneur.2018.00741

**Published:** 2018-09-19

**Authors:** Gregory L. Willis, Jamilee Boda, Christopher B. Freelance

**Affiliations:** The Bronowski Institute of Behavioural Neuroscience, The Bronowski Clinic, Coliban Medical Centre, Kyneton, VIC, Australia

**Keywords:** Parkinson's disease, light therapy, circadian, sleep, depression, anxiety

## Abstract

Parkinson's disease (PD) is a disorder characterized by loss of dopamine (DA) in the nigro-striatal dopamine (NSD) system with the primary symptoms of bradykinaesia, rigidity, tremor, and altered gate. Secondary symptoms including depression, insomnia, involuntary movement, and psychiatric side effects are also commonly observed. While the treatment focus for the past 50 years has been aimed at replacing deficient DA, to relieve the primary symptoms, more recent studies have suggested that the circadian system plays a critical role in the etiology and treatment of this disorder. Several case studies and open label trials have implemented bright light therapy (BT) in an attempt to repair sleep, depression and even the primary motor symptoms of this disorder, however controlled studies are yet to be fully implemented. In this controlled trial, patients that had been maintained on BT daily for 4 months to 5 years previously were assigned to one of three groups: continued polychromatic light, continued with red light or discontinued polychromatic light for a 2 week period. The Movement Disorder Society-Unified Parkinson's Disease Rating Scale (MDSUPDRS), The Parkinson's Disease Questionnaire (PDQ-39), The Beck Depression Inventory II, The Beck Anxiety Inventory, The Epworth Sleep Scale (ESS) and a global rating scale were used to assess patients prior to and at 1 and 2 weeks after commencing the trial. Patients continuing polychromatic BT showed significant improvement on the MDSUPDRS Rating Scale (12 points; *p* = 0.028), the PDQ-39 (10 points; *p* = 0.011), ESS (4 points; *p* = 0.013), and numerous motor and secondary symptoms on a global rating scale. Performance on standardized motor tests also incrementally improved in this group while those exposed to red light and those that discontinued BT treatment deteriorated. These results demonstrate that strategically applied polychromatic light was beneficial in reducing many primary motor and secondary symptoms of PD. Further work investigating the role of light in mitigating PD symptoms and involvement of the circadian system will provide further advances in the treatment of PD.

**Clinical Trial Registration**: http://www.anzctr.org.au, identifier ACTRN12617001309370.

## Introduction

The pharmacological treatment of depression has a long history involving modification of the major neurotransmitters including dopamine (DA), noradrenaline (NA) and serotonin (5HT). It wasn't until 1984 that Rosenthal et al (1) discovered that seasonal affective disorder (SAD) was effectively treatable using bright light therapy (BT). This approach is justified on the basis that it involves the circadian system, as it relates to the natural course of the light/dark (L/D) cycle (2). From this, it might well be surmised that there are different forms of depression, each with their own neuroanatomical and neurochemical substrates. In the case of seasonal affective disorder, the chemistry and underlying anatomy appears to be substantially different from that seen in other forms of depression in that it involves the retino-hypothalamic tract (RHT), the hypothalamus, the pineal and its primary hormone, melatonin (3, 4). The question then arose as to whether BT could be implemented to treat other forms of depression and this approach has been found to be relatively successful (5). In particular, the treatment of Parkinson's disease (PD) patients with bright light has attractive possibilities in that these patients commonly experience drug overdosing and polypharmacy which leads to severe secondary side effects, exacerbation of symptoms and compromise in the efficacy of treatment (6, 7). In this respect the use of BT to counteract the depression and insomnia would serve the important function of reducing the overall drug burden that these patients commonly experience (8). In fact, in several studies examining the effects of light treatment on sleep, depression and other secondary symptoms of PD, this approach has proven to be promising (6, 9, 10). Indeed, there are even studies that have employed light to improve sleep disorders in PD which have found that with this, motor function in PD patients improves as well (11, 12). In spite of this growing background of information there have been two controlled trials with BT in PD with modest treatment outcomes seen in *de novo* patients which improved sleep (11), motor function and quality of life (7). The object of the present trial was to examine the therapeutic benefits of BT in a slightly different population of PD patients compared to those examined previously (7, 11). The first difference is that patients chosen for the present study had been maintained on BT for at least 4 months prior to involvement in the study. This approach was chosen to avoid or minimize technical problems commonly experienced during the initial period of BT that compromise its efficacy. Such intervening variables that hinder a smooth between-subject application of light as an independent variable include poor compliance, uniform positioning, and the onset of drowsiness and these can take several visits to the clinic to overcome. Second, the present trial administered light in the evening, before bedtime, which has been found to be effective in PD patients as these patients are reported to be phase advanced (13–16). On this basis the paradigm for a formal controlled trial was set examining the effects of continued BT compared to the control emission from a light emitting low intensity red light or the discontinuation of BT. It was hypothesized that only those patients in the continuing BT group would experience a maintained therapeutic benefit from the exposure to 1 h of polychromatic light.

## Methods

### Participants

Seventeen male and thirteen female patients diagnosed previously with idiopathic PD were rated at stage I-III on the Hoehn Yahr Scale and had been attending the Bronowski Clinic for a period of time ranging from 4 months to 10 years and had met eligibility criteria were involved in the study. This study was carried out under the guidelines of The National Health and Medical Research Council of Australia (NH&MRC) as defined in “The National Statement on Ethical Conduct in Human Research” (2007) and “The Australia Code for the Responsible Conduct of Research” (2007). This trial was approved and monitored by an independent, *bona fide*, NH&MRC Human Research Ethics Committee from The Swan Research Institute with supplemental ethical standards monitoring provided by The Bronowski Institute Ethical Standards Committee (ESC) throughout the study.

The reasons for ineligibility included, concurrent involvement in another study, patients that were medically complicated, history of psychiatric illness, history of alcohol or narcotic abuse, severe depression or suicidal tendencies, pregnancy, the use of photosensitizing drugs, pre-existing major joint problems, cognitive impairment or focal neurological deficits. Males and females over the age of 45 were eligible. Inclusion criteria required a previous diagnosis of idiopathic PD by a qualified neurologist and receiving DA replacement for at least 12 months and currently undergoing light treatment with compliance at the Bronowski Clinic. The demographics and clinical features of all patients at the commencement of the study are expressed in Table 1.

**Table 1:** Patient Demographics describing the populations for each group employed in the study.

	**Group**		
	**RLG**	**PLG**	**NLG**
Number of males	6	6	5
Number of females	4	4	5
Age range (years)	55–83	54–75	53–79
Average age (years)	70.8	66.9	66.3
Months of BT before trial (range)	5–63	5–61	4–87
Average months of BT before trial	29.9	28.1	45.6
Mean baseline M.D.S.U.P.D.R.S. score ± SD	61.7 ± 30.4	45.0 ± 28.8	56.0 ± 21.7
Mean baseline PDQ-39 score ± SD	26.6 ± 20.0	15.9 ± 10.5	33.3 ± 24.2
Mean baseline BDI-II score ± SD	9.4 ± 7.7	9.0 ± 8.6	11.6 ± 9.1
Mean baseline BAI score ± SD	9.0 ± 7.0	5.6 ± 3.2	12.2 ± 6.6
Mean baseline ESS score ± SD	7.7 ± 3.8	6.1 ± 4.3	8.6 ± 3.4

*The Red Light Group (RLG) served as controls while the Polychromatic Light Group (PLG) was the active group and an additional group served as a comparison after active BT was withdrawn for the duration of the trial (No Light Group; NLG). As a baseline measure to compare the homogeneity between groups a One-Way ANOVA was performed for the 5 measures employed in the trial. There were no significant differences found between the groups for any of the measures used in the study when measured prior to commencing the study during the “PRE” measurement. The obtained scores for the 5 measures are expressed as the mean ± the standard deviation*.

### Apparatus

Light was administered by utilizing a light source containing fluorescent tubes (Apollo BL-6, without ultra-violet emission). The light source was angled from the 11:00 or 1:00 position relative to the sagittal plane of the head. Since melatonin is secreted primarily at night (17, 18) exposure occurred for 1 h between the hours of 20:00 and 22:00 and in most cases this was just prior to retiring and was tied to bedtime. With PD patients having been described as “phase advanced” (13, 14) the time of exposure to light occurred just before the peak in melatonin secretion hypothesized to occur at 22:00–23:00 h. As a general rule of thumb, light was administered at a dose of about 3,000 lux achieved by positioning the device at a distance of about 0.8 to 1 M from the bridge of the nose to the diffuser. All patients had been maintained on BT with polychromatic light prior to commencing the study and were assigned to groups via block randomization into one of three groups: a polychromatic light group (PLG; *n* = 10) serving as the experimental group; a red light group (RLG; *n* = 10) serving as a control group; and a group previously treated with polychromatic light that discontinued their treatment for the 2 week duration of the study (NLG; *n* = 10). For the PLG a slightly frosted gel was used which achieved a < 15% reduction in emission with no barrier to any specific frequency (Lee Filters #420-Light Opal Frost, Y > 85%). Light sources for the RLG were fitted with a red gel to achieve a 90.7% reduction in emission whereby all frequencies below the value of about 575 nm were blocked (Lee Filters #106-Primary /Red, Y = 9.3%). Given that this dramatically reduced the gross emission that patients in this group received the light was moved closer to the patient for the duration of the study (to a distance of 0.8 mm from the bridge of the nose to the diffuser on the light source). The spectral emission of each light source type employed in the study was measured with a spectrometer (JAZ A1382 Spectrometer, Ocean Optics, Florida, USA) and this is shown in Figure 5.

### Patient briefing and consent

There were 2 levels of briefing undertaken regarding the rationale for implementing BT in PD. The admission briefing described the background, efficacy, and mechanism underlying BT and this was done during general admission into the Bronowski Clinic 4 months to 10 years prior to commencing the study. The second briefing was the study briefing itself which was an extension of the admission briefing as it applied to the special modifications of BT required for the study. During the study briefing and prior to commencing each session before the trial started (PRE), at week 1 (W1) and at week 2 (W2), patients were reminded that their participation in the study was completely voluntary, and that they could withdraw at any time and that their decision to withdraw did not affect, in any way, the treatment that they would receive in the future. All patients were given time to read and sign the consent form and were encouraged to ask questions as to the possible risks and benefits of being involved in the study. Patients were informed that there were three groups in the study and that 2 of the groups would have a filter placed over the front of the light to determine if changing the emission would alter any therapeutic benefits the treatment was providing. They were advised that a third group would cease BT for the 2 week duration of the study. They were also informed that they would be placed randomly into one of the 3 treatment groups. Due to the fact that patients had been using BT for some time and incremental therapeutic benefits were frequently reported, it was decided that the study was to be terminated at 2 weeks to minimize the possibility of a major deterioration. Limiting the duration of the study to two weeks would ensure that inherent levels of deception and expectation that are often associated with placebo controlled studies were minimized (19, 20) with a genuine expectation of competent care to serve as equipoise (21).

### Randomization and blinding

When eligibility for study participation was confirmed coded identities for each patient were pooled and then randomly selected in ordered groups of three. The 3 arms of the study were then coded in blocks to represent the 6 possible permutations of order for patient assignment to the 3 groups in the study (e.g., 1,2,3; 1,3,2; 2,1,3; 2,3,1; 3,1,2; 3,2,1). As each group of 3 patients was selected in order, they were assigned to their respective group as dictated by the coded block, which was also randomly selected for each group of 3 patients. Given that all patients admitted to the clinic were placed on light therapy prior to commencing the study this did not bias the process of randomization. Neither the patient selection nor the selection of block assignment were divulged to the clinician performing the assessment until after data analysis was complete at the end of the study. During the briefing it was emphasized that the study was a blinded trial. To achieve this, instructions to patients purposely remained non-directional. They were informed that the aim of the study was to determine whether filtering the light emission from their light source would improve or cause deterioration of their condition. It was also explained that it was important that the clinician remained blinded as to which patient was assigned to which group. Not only was this emphasized at the commencement of the trial but each patient was reminded at the beginning of each subsequent session during the 2 week course of the trial.

### Study design

The present study was a three arm study with each arm implemented to control for intervening variables including patient expectation, the use of the device itself, and the therapeutic value of the emission *per se*. Rather than using *de novo* patients that have never been exposed to BT previously, the present study implemented patients that had undergone BT for a minimal period of 4 months. This design was chosen to minimize or eliminate the technical problems commonly experienced in the use of BT during the initial period of use which compromises its therapeutic efficacy. Such technical problems routinely encountered during this period include positioning of the light source to ensure optimal retinal delivery, strategic time of delivery during the L/D cycle, refusal to compromise competing lifestyle and optimizing compliance. This ensured that application of the independent variable was homogenously applied across all groups in the study (8, 22) but did not bias the randomization process. The first group was the active group (PLG) and was defined as those continuing BT for the 2 week duration of the study. It was hypothesized that this group would maintain or improve on the primary and secondary endpoints during that time. While this group served as the experimental group their expectation from the non-directional briefing would equalize their expectation with that of the control group by the placement of a filter over the light emitting device which, unbeknown to them, only slightly reduced the intensity of the emission but did not compromise its therapeutic potential (< 15%). In this regard, as the emission, *per se*, from the device would remain therapeutic, it was predicted that the group would maintain or exhibit a positive therapeutic response.

The RLG served as a control group and they too had used polychromatic light prior to the study but placed a red filter over the light source at the commencement of the study. This reduced the intensity and limited the broad spectral characteristics of the emission to long wavelengths known to be clinically ineffective (Figure 5). In fact, low intensity red light, similar to that employed in the present study, is routinely implemented as a control procedure in BT studies for SAD and PD (3, 23, 24) and does not readily alter relevant physiological parameters as does polychromatic light. In addition, the expectation of this group was equalized to that of the active group, by providing the same non-directional briefing, informing patients that a change in emission may improve or adversely affect the therapeutic response. It was hypothesized that the RLG would show a decrement in therapeutic response compared to the active group.

The third group served as an additional control group (NLG), and it was hypothesized that they too would demonstrate a reduction in a therapeutic response during the 2 weeks of observation. However, expectation would also play a role in their response in that after several months to years of routine clinical use of BT, they learned that the therapeutic response was incremental requiring 2–3 months of prolonged use before the clinical benefits could be significant (6, 8). Conversely, as with all patients, they knew that discontinuation of BT for short periods of time would also be incremental and loss of therapeutic benefit would be minimal for the duration of the study. Although we hypothesized that therapeutic loss would be minimal over a 2 week period in this group it would be greater than that of the PLG group and similar to that of the RLG. This group, in the context of the RLG, served to control for placement of a light emitting device and for the use of non-specific light exposure, *per se*.

On this basis, the present study implemented what could be regarded as a unique crossover design from long term BT to 3 different groups. The unique feature of this design is that it controlled for the effect of intervening variables common to BT that have not been addressed in similar chronotherapeutic studies undertaken to date (7, 11). Otherwise, the design was similar to that employed in other controlled chronotherapeutic studies examining the effects of BT (11).

### Patient evaluation

During PRE, at the conclusion of the briefing sessions, the following assessments were administered by the attending clinician (GLW): Parts I, II, and IV of the MDSUPDRS, the ESS, the PDQ-39, and the Brief Bronowski Scale (BBS). Part II of the MDSUPDRS, the BDI-II and the BAI were given to the patient to complete after detailed instructions were provided to the patient and carer. In 2 cases more detailed instructions were provided to the patient as they did not readily understand the nature of the task. Clinical assessment of motor performance and psychiatric parameters on the BBS were evaluated as a global rating for each parameter (6, 8). This scale was based on a 1–10 rating with the highest value representing the highest level of symptom severity. The clinical assessment model implemented at the Bronowski Clinic routinely involves the carer/spouse in the assessment procedure and this was encouraged to continue throughout the study. The timed motor test including the elbow to fist (ETF) and the Floor to Knee (FTK) latency have been described in detail previously (8) and were performed at the end of each assessment. Briefly these tests were applied as follows: In the ETF latency the upper arm is extended and held 90° to the plane of the body with the lower arm again extended upward 90° to the upper arm. The top of the clenched fist points toward the ceiling with clenched fingers orientated toward the patient's face. With the opposite hand open, the patient begins by placing the palm of his hand on the top of the clenched fist. When told to begin, the patient brings the open hand down, twisting it 180° and then touching the elbow from underneath. This test measures upper limb dexterity, control, coordination and strength. The latency to perform this task 10 times is recorded. In addition, the quality of performance using force, speed, hand placement and task completion were also noted. In the FTK latency, the patient stands erect with their weight suspended on one foot. The other foot is then raised off the floor touching the inside of the leg just below the knee with the side of the foot. The foot is then returned to the floor. The time required to perform this task 10 consecutive times is recorded. The quality of performance and the need for additional support to maintain balance is also recorded. For both tests, the patient was required to count out loud as each of the 10 subunits is completed. All tests were applied during the light cycle between the hours of 09:00 and 16:00 h. All patients returned to their routine use of polychromatic light at the end of the study.

Signs and symptoms monitored included bradykinaesia, rigidity, tremor (left), tremor (right), walking, dyskinaesia, masked face, balance, speech, depression, agitation/ anxiety, sleep (total hours, number of awakenings, do they readily fall back to sleep, number of day naps, involuntary movement, dreaming, medication, and compliance). All clinical parameters on the BBS were scored as absent (0), slight (1–3), moderate (4–6), or severe (7–10), on a modified Likert Scale similar to that reported previously (25) with the score of the pre-score session serving as the baseline. Relative improvements or degradations during W1 and W2 assessments were scaled in regard to performance on the previous session. Time of the last medication and on-off status of each patient was recorded during each assessment. Results using this scale have been published previously (6, 8).

### Statistical analysis

Descriptive statistics regarding gender, age, medication, age at diagnosis, duration of phototherapy, duration of disease with the mean, standard deviation and range were expressed in tabular form. For all dependent variables, median, mean, 1st, 2nd, 3rd and 4th quartile, standard deviation and standard error were calculated. Statistical analysis for all dependent variables was undertaken using the IBM SPSS Statistics 24 Software for Windows Package implementing preliminary analysis with a general linear model ANOVA to examine within and between group factors. However, due to frequent occurrence of skewed distribution, non-homogenous variance, large standard deviations and the low number of subjects per group, which violate basic assumptions of parametric testing, non-parametric tests were chosen to evaluate differences in performance between groups, over time, to minimize type I and type II errors. Two patients were removed from the study due to adverse effects and the data from a third patient was not used for analysis as they accidentally revealed their treatment to the assessing clinician during assessment on W1. This left a total of 9 patients per group for final analysis. On this basis, the Related Samples Friedman's Two-Way ANOVA by Ranks (SFAR) was first performed to detect any main effect examining differences in performance between PRE, serving as the baseline, compared to W1 and W2 for each of the 3 groups employed in the study. To determine where the significant differences lay the Related Samples Wilcoxon Signed Rank Test (RSWSRT) between PRE and W1, PRE and W2, and W1 and W2 were performed. The confidence levels were chosen *a priori* and set at 5% to depict a significant effect while confidence levels ranging from 0.051 to 0.099 depicted a trend.

The working hypothesis was constructed around the assumption that a heterogeneous sample would best serve to answer the question as to the potential efficacy of BT in PD. For this reason a maximum variation sample was used in the present study as the time since diagnosis or disease severity had not been shown in a previous case series study (6), open label retrospective (8) and controlled studies (7, 11) to impair the therapeutic response to BT. Nevertheless, a one-way ANOVA was performed for scores on the 5 major assessments comparing the sample across all 3 arms during the PRE phase of the study. This was done to determine if the samples varied at baseline prior to application of the independent variables. Results from the analysis revealed that overall baseline scores for the PLG, the RLG and the NLG did not differ significantly for the MDSUPDRS (*df* = 2, 26; *F* = 0.872, *p* = 0.431), the PDQ-39 (*df* = 2, 26; *F* = 1.905, *p* = 0.171), the BDI-II (*df* = 2, 26; *F* = 0.232, *p* = 0.795), the BAI (*df* = 2, 26; *F* = 2.936, *p* = 0.072), and the ESS (*df* = 2, 26; *F* = 0.942, *p* = 0.404) suggesting that samples in the 3 groups were homogenous.

## Results

**Figure 1:**
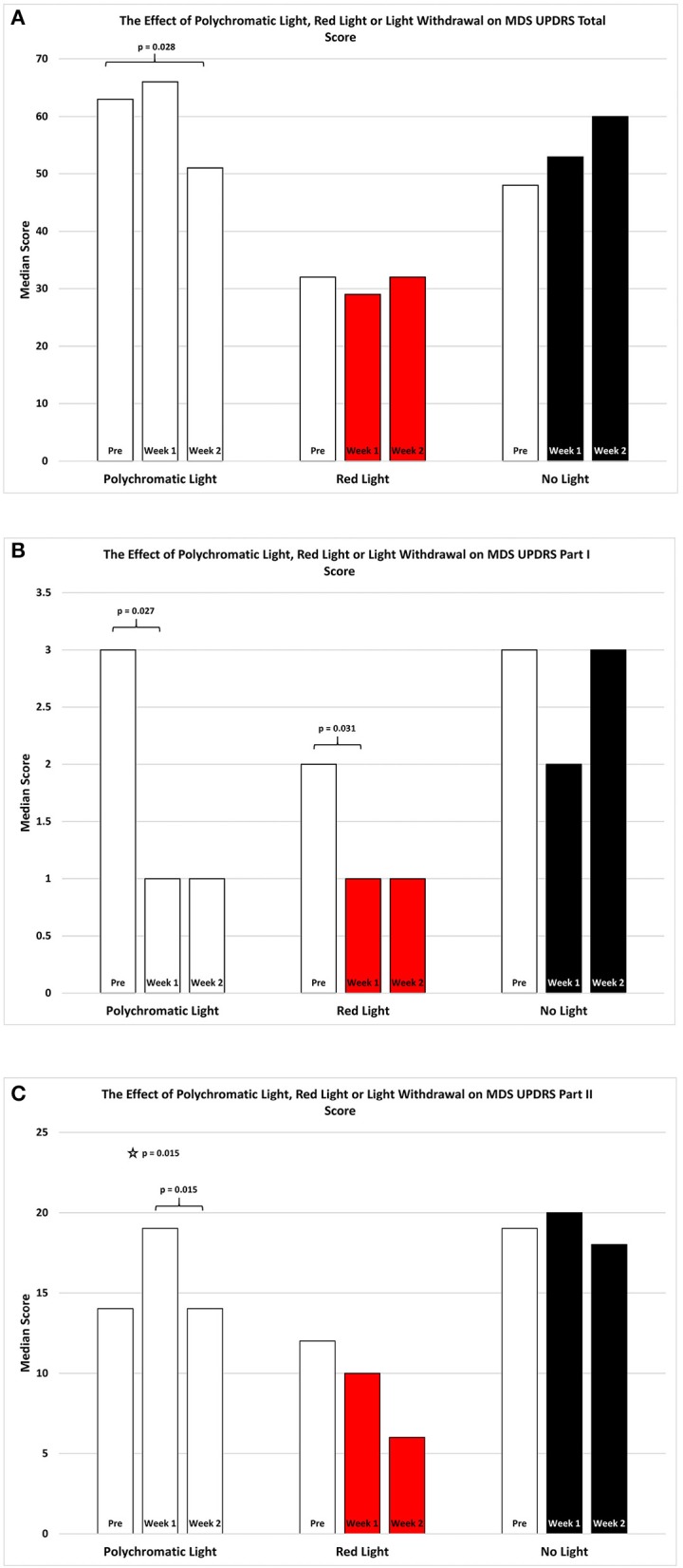
The effect of continued daily exposure to polychromatic light, red light or withdrawal of polychromatic light on M.D.S.U.P.D.R.S. scores in patients treated previously with BT. Performance on the Total Score **(A)**, Part I **(B)** and Part II **(C)** is expressed as the median score for each group after 3 assessments over a 2 week period. The pre-score served as the baseline and was obtained when the study commenced and before patients were randomly assigned to their respective treatment groups. The inclusion bars show significant statistical comparisons with *p-*values indicating the levels of significance obtained. Statistical comparisons were made between baseline (Pre) and Week 1, Pre and Week 2 and Week 1 and Week 2. Significant levels were determined *a priori* with a significant effect defined as *p* ≤ 0.05 while trends were designated as *p*-values ranging from 0.051 to 0.099. The star represents a significant overall effect using the Friedman's Two Way ANOVA while the inclusion bars mark significant comparisons using the Wilcoxon Signed Rank Test.

As shown in Figure 1A the effect of BT on total MDSUPDRS Score over the 2 weeks of observation was significantly improved by the second week only in the PLG. SFAR revealed a trend (*p* = 0.074) in this group. Further analysis revealed that while the median score changed from 63 (Q1 = 41.5; Q3 = 74) to 66 (Q1 = 30; Q3 = 69.5) after the first week of daily exposure this change was not significant (RSWSRT, *p* = 0.528). However, the median score of this group during the second week (Q1 = 31; Q3 = 62) dropped to 51 which was improved compared to the pre-score and this was significant (RSWSRT, *p* = 0.028). The performance of the RLG patients and the NLG did not show significant change in their overall scores at any time during the 2 weeks of observation.

As shown in Figure 1B, the effect of light exposure on Part 1 of the MDSUPDRS, over the 2 weeks of treatment SFAR revealed a trend for the RLG (*p* = 0.055) to show improvement. RSWSRT revealed significant improvement in both the PLG and RLG groups between the PRE and W1 measurements only. In these cases the median score changed from a raw score of 2 to 1 on this parameter for the RLG (PRE, Q1 = 0.5; Q3 = 4.5 vs. W2; Q1 = 0; Q3 = 2.5, *p* = 0.031; RSWSRT). For the PLG there was also significant improvement during W1 vs. the pre-test score (PRE, Q1 = 1.0; Q3 = 4.5 vs. W1; Q1 = 0.0; Q3 = 3.0; *p* = 0.027, RSWSRT, with the score improving from 3 to 1 points on the daily living subscale. However, there was no further change on the score for this group comparing performance on pre-test measurements vs. W2 (Q1 = 0; Q2 = 4, *p* = 0.102). Similarly, there was no difference between the scores on W1 vs. W2 for this parameter (RSWSRT; *p* = 0.160). While differences were observed between the pre-treatment score, the W1 score and the W2 score for subjects in the NLG, none of these were significant (SFAR; Median pre-test = 3.0; Q1 = 2.0; Q3 = 3.5: W1 Median PRE = 2.0; Q1 = 1.0; Q3 = 5.0: Median W2 = 3.0; Q1 = 1.0; Q3 = 6.0, *p* = 0.687).

Figure 1C illustrates the changes in score on Part II of the MDSUPDRS. While the patients in the RLG did not show a significant improvement on the first or second week after daily light treatment (W1; Median = 10; Q1 = 4; Q3 = 19.5: W2; Median = 6; Q1 = 5; Q3 = 19.5; SFAR, *p* = 0.293), those in the PLG group showed an overall significant change during the course of treatment (SFAR; *p* = 0.015). Further analysis using RSWSRT revealed that the significant difference was between PRE and W2 for the PLG (Median PRE = 14.0; Q1 = 11.0; Q3 = 31.0: Median W2 = 14.0; Q1 = 6.5; Q3 = 5.0: Median W2 = 3.0; Q1 = 1.0; Q3 = 29.0, *p* = 0.015), while their performance during W1 was not significantly different from their PRE score (Median = 19.0; Q1 = 6.0; Q3 = 29.0, *p* = 0.108). Those patients receiving no light during the 2 weeks of observation did not show a significant change in their performance on Part II of the MDSUPDRS (SFAR, *p* = 0.690). In part IV of the MDSUPDRS there was a trend revealed for the main effect in the RLG (SFAR, 0.223; *p* = 0.095), with further examination revealing a weak trend between PRE and W1 (RSWSRT, *p* = 0.084). No significant changes in performance over the 2 week period were seen in the PLG or NLG groups. Similarly, there were no significant changes in Part III of the MDSUPDRS Table 2.

**Table 2:** The effect of continued daily exposure to polychromatic light, red light or withdrawal of therapeutic light on parameters showing no change in patients that had used the light for at least 4 months prior to commencing the study.

**Assessment**	**Comparison**	**Median Pre**	**Q1 Pre**	**Q3 Pre**	**Median Week 1**	**Q1 W1**	**Q3 W1**	**Median Week 2**	**Q1 W2**	**Q3 W2**	***P*-value**
UPDRS III	Poly within	35.0	20.0	53.5	32.0	21.0	55.5	28.0	23.0	46.0	0.539
	Red within	25.0	9.5	48.5	17.0	16.5	36.5	22.0	9.5	45.5	0.459
	No light within	27.0	21.0	42.5	36.0	20.5	40.5	36.0	23.0	40.0	0.264
UPDRS IV	Poly within	0.0	0.0	0.0	0.0	0.0	3.5	0.0	0.0	4.5	0.223
	Red within	1.0	0.0	2.5	2.0	0.0	5.5	0.0	0.0	4.0	0.095*
	No light within	0.0	4.0	5.0	1.0	0.0	5.5	2.0	0.0	3.5	0.084*
PDQ-39 ADL	Poly within	3.0	2.5	6.5	3.0	1.5	7.0	2.0	1.0	7.5	0.267
	Red within	1.0	0.5	5.0	1.0	0.0	2.5	1.0	0.0	5.0	0.494
	No light within	4.0	2.5	5.5	2.0	2.0	4.0	2.0	1.5	6.5	0.207
PDQ-39 Social	Poly within	0.0	0.0	0.5	0.0	0.0	0.5	0.0	0.0	0.0	0.670
	Red within	0.0	0.0	0.5	0.0	0.0	1.0	0.0	0.0	0.5	0.607
	No light within	0.0	0.0	4.0	0.0	0.0	2.5	0.0	0.0	0.5	0.113

Values are expressed as the median scores for each group tested, for each of the 3 times assessed over a 2 week period. The pre-score served as the baseline and was obtained when the study commenced and before patients were randomly assigned to their respective treatment groups. The (Q1) and third (Q3) quartile values show the deviation of scores around the median and are expressed for each parameter. Significant changes between the two medians tested and trends between those groups are highlighted with gray highlight and an asterisk (*). The trends were defined as p values ranging from 0.051 to 0.099 with these levels chosen a priori.

**Figure 2:**
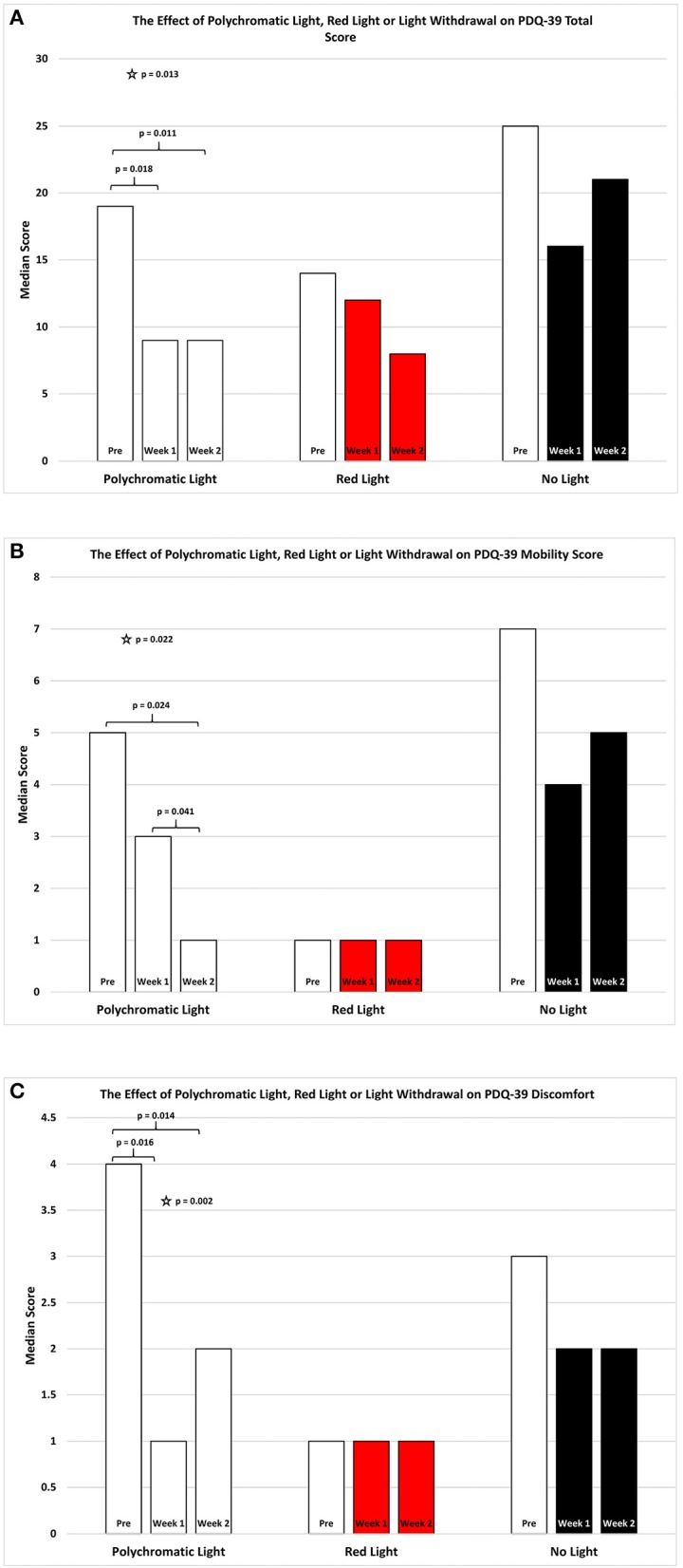
The effect of continued daily exposure to polychromatic light, red light or withdrawal of polychromatic light on the Total Score **(A)**, Mobility **(B)** and Discomfort **(C)** sub-sections of the PDQ-39 is expressed as the median score for each group after 3 assessments over a 2 week period. The pre-score served as the baseline and was obtained when the study commenced and before patients were randomly assigned to their respective treatment groups. The inclusion bars show significant statistical comparisons with *p*-values indicating the levels of significance obtained. Statistical comparisons were made between baseline (Pre) and Week 1, Pre and Week 2 and Week 1 and Week 2. Significant levels were determined *a priori* with a significant effect defined as *p* ≤ 0.05 regarded while trends were designated as *p* values ranging from 0.051 to 0.099. The star represents a significant overall effect using the Friedman's Two Way ANOVA while the inclusion bars mark significant comparisons using the Wilcoxon Signed Rank Test.

As shown in Figure 2A there was an improvement in the total PDQ39 score for the patients continued on polychromatic light and this was highly significant (SFAR, *p* = 0.013). Comparison specifically between PRE and W1 were also significant (Median = 19 vs. 9; Q1 = 11.5 vs. 6.5; Q3 = 41 vs. 35.5, *p* = 0.018) as was the comparison between PRE and W2 (RSWSRT, *p* = 0.160 pre Median = 19; Q1 = 11.5, Q3 = 41; W1 Median = 9; Q1 = 6.5; Q3 = 35.5, *p* = 0.011). There was also a weak trend between W1 vs. W2 in these patients (RSWSRT, *p* = 0.160 Medians, Q1 and Q3 values states above; *p* = 0.096). Red light exposure for a 2 week period had little or no effect upon the total score of the PDQ-39 assessment (SFAR: Median for PRE = 14; W1 = 12; W2 = 8: Q1 for PRE = 7.5; W1 = 4.5; W2 = 4: Q3 for PRE = 24; W1 = 15; W2 = 15.5, *p* = 0.244). Similarly, withdrawing light from patients was without significant effect (SFAR; Median for PRE = 25; W1 = 16; W2 = 21: Q1 for PRE = 18; W1 = 12; W2 = 9: Q3 for PRE = 46; W1 = 35; W2 = 35, *p* = 0.123).

As shown in Figure 2B there was an overall improvement in the mobility subsection of the PDQ39 score for the patients continued on polychromatic light and the difference was also highly significant (SFAR, *p* = 0.022). Comparison between PRE and W1 for the PLG maintained on light showed a weak trend (Median = 5 vs. 3; Q1 = 1 vs. 1; Q3 = 4.1 vs. 5.5, RSWSRT, *p* = 0.085) while the comparison between PRE and W2 was highly significant (RSWSRT, *p* = 0.160; pre Median = 5; Q1 = 1, Q3 = 8; W2: Median = 1; Q1 = 0; Q3 = 5.5, *p* = 0.024). There was also a significant difference between W1 vs. W2 (Medians, Q1 and Q3 values stated above, RSWSRT, *p* = 0.160, *p* = 0.041). Red light exposure for a 2 week period again had little or no effect upon the mobility score of the PDQ-39 assessment (SFAR, Median for PRE = 1; W1 = 1; W2 = 1: Q1 for PRE = 1; W1 =; W2 = 0: Q3 for PRE = 29; W1 = 5; W2 = 4, *p* = 0.236). Similarly, withdrawing light from patients was without significant effect (SFAR, Median for PRE = 7; W1 = 4; W2 = 5: Q1 for PRE = 2; W1 = 0; W2 = 2: Q3 for PRE = 10; W1 = 10; W2 = 15, *p* = 0.163.

As shown in Figure 2C there was an overall improvement in the discomfort subsection of the PDQ39 score for the PLG and the difference was also highly significant (Friedman's Two Way ANOVA, *p* = 0.002). Comparison between PRE and W1 for the group maintained on light showed a significant improvement (Median = 4 vs. 1; Q1 = 1 vs. 0; Q3 = 5 vs. 4.5, RSWSRT, *p* = 0.016) while the comparison between PRE and W2 was highly significant (RSWSRT; PRE Median = 4; Q1 = 1, Q3 = 5; W2; Median = 2; Q1 = 0; Q3 = 4.5, *p* = 0.014). There was no significant difference between W1 vs. W2 (Medians, Q1 and Q3 values stated above, RSWSRT, *p* = 0.414). Red light exposure for a 2 week period again had little or no effect upon the discomfort score of the PDQ-39 assessment (SFAR, Median for PRE = 1.0; W1 = 1; W2 = 1: Q1 for PRE = 0; Week 0 = W2 = 0: Q3 for PRE = 2.5; W1 = 2; W2 = 1.5, *p* = 0.810). Similarly, withdrawing light from patients was without significant effect (SFAR: Median for PRE = 3; W1 = 2; W2 = 2: Q1 for PRE = 2; W1 = 0; W2 = 1: Q3 for PRE = 5.5; W1 = 4.5; W2 = 5, *p* = 0.355).

**Figure 3:**
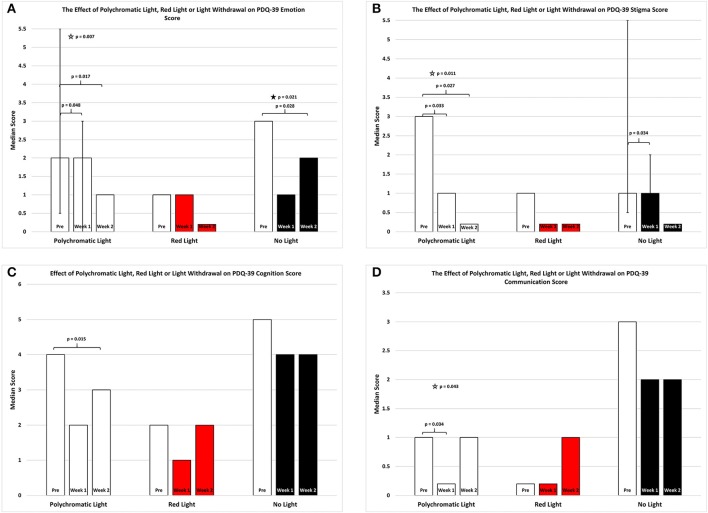
The effect of continued daily exposure to polychromatic light, red light or withdrawal of polychromatic light on the Emotion **(A)**, Stigma **(B)**, Cognition **(C)**, and Communication **(D)** sub-sections of the PDQ-39 is expressed as the median score for each group after 3 assessments over a 2 week period. The pre-score served as the baseline and was obtained when the study commenced and before patients were randomly assigned to their respective treatment groups. The inclusion bars show significant statistical comparisons with *p*-values indicating the levels of significance obtained. Statistical comparisons were made between baseline (Pre) and Week 1, Pre and Week 2 and Week 1 and Week 2. Significant levels were determined *a priori* with a significant effect defined as *p* ≤ 0.05 while trends were designated as *p* values ranging from 0.051 to 0.099. The star represents a significant overall effect using the Friedman's Two Way ANOVA while the inclusion bars mark significant comparisons using the Wilcoxon Signed Rank Test. The T-bars represent first quartile and third quartile values. This provides a reference point whereby the range of values around the median serves to assist in visualizing the direction of change between medians in instances where median values were equal but significantly different.

As shown in Figure 3A there was an overall improvement in the emotion subsection of the PDQ39 score for the patients continued on polychromatic light and the difference was also highly significant (SFAR, *p* = 0.007). Comparison between PRE and W1 for the group maintained on light showed a weak trend (Median = 2 vs. 2; Q1 = 0.5 vs. 0.0; Q3 = 5. vs. 3.0, RSWSRT, *p* = 0.048) while the comparison between PRE and W2 was highly significant (RSWSRT, PRE Median = 2.0; Q1 = 0.05, Q3 = 5.5; W2, Median = 1.0; Q1 = 0.0; Q3 = 3.0, *p* = 0.017). There was also a weak trend between W1 vs. W2 (Medians, Q1 and Q3 values stated above, RSWSRT, *p* = 0.085). It is important to note that while the median scores for the PRE group and the W1 group were equal, the direction of the significant change can be determined by the interquartile range expressed for each time period examined. Red light exposure for a 2 week period again had little or no effect upon the mobility score of the PDQ-39 assessment (SFAR, Median for PRE = 1.0; W1 = 1.0; W2 = 0.0: Q1 for PRE = 0.5; W1 = 0.5; W2 = 0.0: Q3 for PRE = 0.4; W1 = 4.5; W2 = 3.0, *p* = 0.2). Withdrawing light from patients, however, produced an overall significant effect (SFAR: Median for PRE = 3.0; W1 = 1.0; W2 = 2.0: Q1 for PRE = 2.5; W1 = 1.5; W2 = 0.0: Q3 for PRE = 10.5; W1 = 8.5; W2 = 4.0, *p* = 0.021). To determine where this effect lay, comparison between PRE and W1 revealed a weak trend (RSWSRT, *p* = 0.082) while the change between PRE and W2 was highly significant (RSWSRT, *p* = 0.028). There was no difference between W2 and W3 (RSWSRT, *p* = 0.395).

Figure 3B depicts an overall improvement in the stigma subsection of the PDQ-39 score for the patients continued on polychromatic light and the difference was highly significant (SFAR, *p* = 0.011). Comparison between PRE and W1 for the group maintained on light showed a significant improvement (Median = 3.0 vs. 1.0; Q1 = 0.0 vs. 0.0; Q3 = 6.0 vs. 3.5; RSWSRT, *p* = 0.033) while the comparison between PRE and W2 was highly significant (RSWSRT, PRE Median = 3.0; Q1 = 0.0; Q3 = 6.0: W2 Median = 0.0; Q1 = 0.0; Q3 = 1.0, *p* = 0.027). There was no significant difference between W1 vs. W2 (Medians, Q1 and Q3 values stated above; RSWSRT, *p* = 0.102). Red light exposure for a 2 week period again had little or no effect upon the stigma score of the PDQ-39 assessment (SFAR, Median for PRE = 1.0; W1 = 0.0; W2 = 0.0: Q1 for PRE = 0.0; W1 = 0.0; W2 = 0.0: Q3 for PRE = 2.5; W1 = 1.5; W2 = 1.0, *p* = 0.268). Withdrawing light from patients, however, produced a strong trend of improvement (SFAR, Median for PRE = 1.0; W1 = 1.0; W2 = 0.0: Q1 for PRE = 0.5; W1 = 0.0; W2 = 0.0: Q3 for PRE = 5.5; W1 = 2.0; W2 = 1.5, *p* = 0.065). To determine where this effect lay comparison between PRE and W1 revealed a significant improvement (RSWSRT, *p* = 0.034) while the change between PRE and W2 was not significant (RSWSRT, *p* = 0.157). It is important to again note that while PRE and W1 the median scores were equal, the direction of the change in this instance was determined by the interquartile range and this clearly shows that the values decreases from PRE to W1 for the NLG.

Figure 3C depicts an overall improvement in the cognition subsection of the PDQ39 score for the patients continued on polychromatic light and this was a strong trend bordering on significance (SFAR, *p* = 0.053). Further comparison between PRE and W1 for this group did not demonstrate a significance change (Median = 4.0 vs. 2.0; Q1 = 2.1 vs. 1.0; Q3 = 5.5 vs. 4.5, RSWSRT, *p* = 0.135) while the comparison between PRE and W2 was significant (RSWSRT, pre Median = 4.0; Q1 = 2.0, Q3 = 5.5; W2, Median = 3.0; Q1 = 0.5; Q3 = 4.5, *p* = 0.015). There was no significant difference between W1 vs. W2 (Medians, Q1 and Q3 values stated above; RSWSRT, *p* = 0.796). Red light exposure for a 2 week period again had little or no effect upon the cognition score of the PDQ-39 assessment (SFAR, Median for PRE = 2.0; W1 = 1.0; W2 = 2.0: Q1 for PRE = 1.5; W1 = 0.5; W2 = 0.0: Q3 for PRE = 3.0; W1 = 2.5; W2 = 3.0; *p* = 0.687). Similarly, withdrawing light from patients was without significant effect (SFAR, Median for PRE = 5.0; W1 = 4; W2 = 4.0: Q1 for PRE = 2.5; W1 = 2.0; W2 = 2.5: Q3 for PRE = 6.0; W1 = 4.0; W2 = 4.5, *p* = 0.679).

Figure 3D depicts the changes in the communication subsection of the PDQ-39 score for the patients continued on polychromatic light and there was a reduction in this score for this group which was significant (SFAR, *p* = 0.043). Further comparison between PRE and W1 for this group revealed a significant change (Median = 1.0 vs. 0.0; Q1 = 0.5 vs. 0.0; Q3 = 2.0 vs. 1.0, RSWSRT, *p* = 0.034) while the comparison between PRE and W2 demonstrated a strong trend (RSWSRT, PRE Median = 1.0; Q1 = 0.5; Q3 = 2.0; W2, Median = 1.0; Q1 = 0.0; Q3 = 1.5, *p* = 0.059). There was no significant difference between W1 vs. W2 (Medians, Q1 and Q3 values stated above; RSWSRT, *p* = 0.655).

Red light exposure for a 2 week period again had little or no effect upon the communication score of the PDQ-39 assessment (SFAR, Median for PRE = 0.0; W1 = 0.0; W2 = 1.0: Q1 for PRE = 0.0; W1 = 0.0; W2 = 0.0: Q3 for PRE = 3.0; W1 = 2.0; W2 = 1.5, *p* = 0.790). Similarly, withdrawing light from patients was without significant effect (SFAR, Median for PRE = 3.0; W1 = 2.0; W2 = 2.0: Q1 for PRE = 1.5; W1 = 0.5; W2 = 0.5: Q3 for PRE = 5.5; W1 = 4.0; W2 = 4.5, *p* = 0.228).

**Figure 4:**
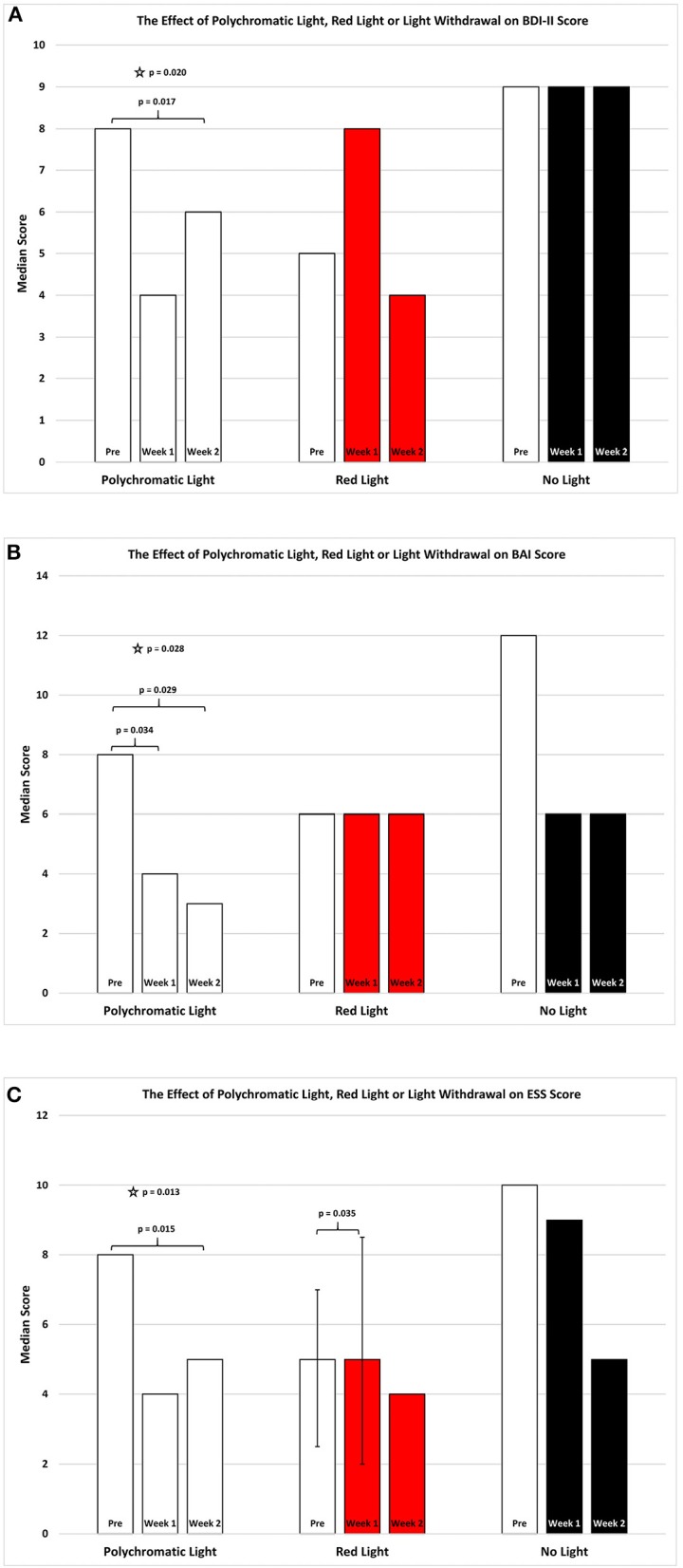
The effect of continued daily exposure to polychromatic light, red light or withdrawal of polychromatic light on the BDI-II **(A)**, BAI **(B)**, and the ESS **(C)** is expressed as the median score for each group after 3 assessments over a 2 week period. The pre-score served as the baseline and was obtained when the study commenced and before patients were randomly assigned to their respective treatment groups. The inclusion bars show significant statistical comparisons with *p*-values indicating the levels of significance obtained. Statistical comparisons were made between baseline (Pre) and Week 1, Pre and Week 2 and Week 1 and Week 2. Significant levels were determined *a priori* with *p* ≤ 0.05 regarded as significant while trends were designated as *p*-values ranging from 0.051 to 0.099. The star represents a significant overall effect using the Friedman's Two Way ANOVA while the inclusion bars mark significant comparisons using the Wilcoxon Signed Rank Test.

Figure 4A shows the change in score on BDI-II for the three treatment groups in the study. For patients continuing with daily polychromatic light exposure there was a significant overall reduction in this score for this group which was not seen in the other groups (SFAR, *p* = 0.020). In depth comparison between PRE and W1 for this group revealed a strong trend toward reduced depression (Median = 8.0 vs. 4.0; Q1 = 0.0 vs. 1.5; Q3 = 16.0 vs. 14.5, RSWSRT, *p* = 0.058) while the comparison between PRE and W2 demonstrated a significant improvement in depressive state (RSWSRT, PRE Median = 8.0; Q1 = 0.0; Q3 = 16.0: W2, Median = 6.0; Q1 = 2.0; Q3 = 10.0, *p* = 0.017). There was no significant difference between W1 vs. W2 (Medians, Q1 and Q3 values stated above, RSWSRT, *p* = 0.443). Red light exposure for a 2 week period again had little or no effect upon the BDI-II score (SFAR, Median for PRE = 5.0; W1 = 8.0; W2 = 4.0: Q1 for PRE = 4.5; W1 = 1.0; W2 = 1.0: Q3 for PRE = 13.0; W1 = 11.0; W2 = 13.0, *p* = 0.422). Similarly, withdrawing light from patients was without significant effect (SFAR, Median for PRE = 9.0; W1 = 9.0; W2 = 9.0: Q1 for PRE = 4.5; W1 = 3.5; W2 = 4.0: Q3 for PRE = 18.5; W1 = 16.5; W2 = 14.0, *p* = 0.343).

**Figure 5:**
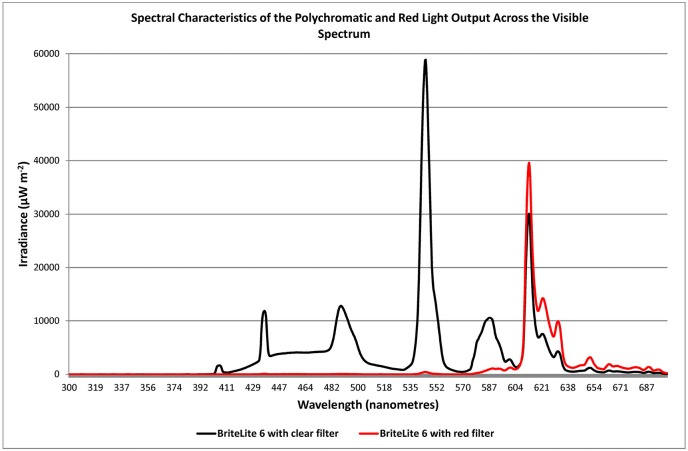
Irradiance spectra of the emission from the BL6 light source after filters were fitted resulting in modification of polychromatic emission with slightly translucent (full spectrum, < 15% intensity reduction; Lee Filters #420-Light Opal Frost, Y > 85%; black trace) or primary red emission filter (90.7% reduction in emission of all frequencies below the value of about 575 nm; Lee Filters #106-Primary Red, Y = 9.3%; red trace).

Figure 4B shows the change in score on the BAI for the three treatment groups. When daily polychromatic light exposure was continued there was a significant overall reduction in this score for this group and this was not seen in the other groups (SFAR, *p* = 0.028). Further comparison between PRE and W1 for this group revealed a strong, significant effect depicting a reduction in the depressive state (Median = 8.0 vs. 4.0; Q1 = 3.0 vs. 2.5; Q3 = 14.5 vs. 6.5, RSWSRT, *p* = 0.034) while the comparison between PRE and W2 demonstrated a further significant improvement in depression (RSWSRT, PRE Median = 8.0; Q1 = 3.0, Q3 = 14.5: W2 Median = 3.0; Q1 = 2.5; Q3 = 7.5, *p* = 0.029). There was no significant difference between W1 vs. W2 (Medians, Q1 and Q3 values stated above; RSWSRT, *p* = 0.104).

Figure 4C depicts the changes in the scores on the Epworth Sleep Scale for the patients continued on polychromatic light and there was a reduction in this score for this group which was significant (SFAR, *p* = 0.013). Detailed comparison between PRE and W1 for this group revealed a trend toward improvement (Median = 8.8 vs. 4.0; Q1 = 4.0 vs. 2.0; Q3 = 2.0 vs. 10.0 vs. 8.5, RSWSRT, *p* = 0.090) while the comparison between PRE and W2 demonstrated a highly significant improvement (Median = 8.0 vs. 5.0; Q1 = 4.0 vs. 1.5; Q3 = 2.0 vs. 10.0 vs. 9.5, RSWSRT, *p* = 0.015). There was no significant difference between W1 vs. W2 (Medians, Q1 and Q3 values stated above, RSWSRT, *p* = 0.435). Red light exposure for a 2 week period revealed an overall weak trend toward worsening on the ESS (SFAR, Median for PRE = 5.0; W1 = 5.0; W2 = 4.0: Q1 for PRE = 2.5; W1 = 2.0; W2 = 2.5: Q3 for PRE = 7.0; W1 = 8.5; W2 = 9.5, *p* = 0.088). Further analysis revealed that the difference between the two time periods of PRE vs. W1 were significant (RSWSRT, *p* = 0.035), while the differences between PRE and W1 and W1 and W2 were not (RSWSRT, *p* = 0.015 and *p* = 0.340 and *p* = 0.317, respectively). Withdrawing light from patients was without significant effect on the ESS score (SFAR, Median for PRE = 10.0; W1 = 9.0; W2 = 5.0: Q1 for PRE = 5.0; W1 = 6.0; W2 = 4.0: Q3 for PRE = 11.5; W1 = 10.5; W2 = 9.5, *p* = 0.625).

The changes in score on the timed motor test, ETF Latency for the right arm in the three treatment groups varied only slightly during the 2 weeks of modified light exposure. When daily exposure to polychromatic light was continued there was a weak trend for this group to reduce the performance time and this was not seen in the other groups (SFAR, *p* = 0.053). While continued exposure to polychromatic light for 2 weeks did not significantly change the time required to perform this task in the comparison between PRE and W1 (SFAR, *p* = 0.641) the comparison between PRE and W2 demonstrated a small but significant improvement (RSWSRT; (Median = 9.5 vs. 9.5; Q1 = 8 .25 vs. 8.0; Q3 = 11.46 vs. 11.3, *p* = 0.05). There was also a weak trend in the difference between W1 vs. W2 (RSWSRT, Median = 10.0 vs. 9.5; Q1 = 8.35 vs. 8.0; Q3 = 11.45 vs. 11.3, *p* = 0.093) revealing an improvement of about 0.5 s.

Red light exposure for a 2 week period again had little or no effect upon the ETF latency (SFAR, Median for PRE = 8.9; W1 = 9.6; W2 = 9.0: Q1 for PRE = 8.3; W1 = 8.3; W2 = 8.8: Q3 for PRE = 10.25; W1 = 11.65; W2 = 10.6, *p* = 0.641). Similarly, withdrawing light from patients was without significant effect (SFAR, Median for PRE = 8.7; W1 = 8.6; W2 = 8.9: Q1 for PRE = 7.9; W1 = 8.0; W2 = 7.8: Q3 for PRE = 10.0; W1 = 9.8; W2 = 10.5, *p* = 0.895). Note again that for the PRE vs. W2 comparison the median scores were equal. Therefore, the direction of the change was determined by the interquartile range and this clearly illustrates that the latency decreased in the vicinity of 0.16–0.25 s from PRE to W1.

Similarly, the latency to perform the floor to knee latency for the right leg in the three treatment groups were only slightly affected. When daily exposure to polychromatic light was continued there was a significant reduction in the performance time and this was not seen in the other groups (SFAR, *p* = 0.027). Exposure to polychromatic light for 2 weeks did not significantly change the time required to perform this motor task in the comparison between PRE and W1 (RSWSRT, *p* = 0.722) but the comparison between PRE and W2 demonstrated a significant reduction in the FTK latency (RSWSRT, Median = 10.5 vs. 10.3; Q1 = 9.1 vs. 8.0; Q3 = 12.6 vs. 12.0, *p* = 0.036). There was also a weak trend in the difference between W1 vs. W2 (RSWSRT, Median = 10.6 vs. 10.3; Q1 = 8.8 vs. 8.0; Q3 = 11.55 vs. 12.0, *p* = 0.086) revealing a very minor, incremental improvement. Red light exposure for a 2 week period again had little or no effect upon the ETF latency (SFAR, Median for PRE = 9.5; W1 = 8.9; W2 = 8.7: Q1 for PRE = 8.5; W1 = 8.2; W2 = 8.1: Q3 for PRE = 10.25; W1 = 11.65; W2 = 10.6, *p* = 0.703). Similarly, withdrawing light from patients was without significant effect (SFAR, Median for PRE = 8.7; W1 = 8.6; W2 = 8.9: Q1 for PRE = 7.9; W1 = 8.0; W2 = 7.8: Q3 for PRE = 10.9; W1 = 11.65; W2 = 11.8, *p* = 0.247).

When floor to knee latency values for both legs combined were analyzed together minor changes between the three treatment groups were observed. When daily exposure to polychromatic light was continued there was a significant reduction in the performance time and this was not seen in the other groups (SFAR, *p* = 0.036). Further analysis reveals that exposure to polychromatic light for 2 weeks did not significantly change the time required to perform this motor task in the comparison between PRE and W1 (RSWSRT, *p* = 0.383) but the comparison between PRE and W2 demonstrated a trend in reducing the FTK latency (RSWSRT, Median = 10.6 vs. 10.0; Q1 = 9.6 vs. 8.3; Q3 = 12.5 vs. 12.5, *p* = 0.058). There was no significant difference between W1 vs. W2 (RSWSRT, *p* = 0.306). Red light exposure for a 2 week period again had little or no effect upon the ETF latency (SFAR, Median for PRE = 9.45; W1 = 8.95; W2 = 8.85: Q1 for PRE = 8.7; W1 = 8.5; W2 = 8.2: Q3 for PRE = 11.0; W1 = 11.3; W2 = 12.2, *p* = 0.202). Similarly, withdrawing light from patients was without significant effect (SFAR, Median for PRE = 9.9; W1 = 9.8; W2 = 9.05: Q1 for PRE = 8.2; W1 = 8.9; W2 = 8.1: Q3 for PRE = 10.2; W1 = 10.3; W2 = 9.8, *p* = 0.214).

Scores for the bradykinaesia scale on the BBS varied minimally across the three treatment groups. When daily exposure to polychromatic light was continued there was a weak trend in the bradykinaesia score (SFAR, *p* = 0.097). Further analysis reveals that exposure to polychromatic light for 2 weeks did not significantly change the bradykinaesia score in the comparison between PRE and W1 (RSWSRT, *p* = 0.157) but the comparison between PRE and W2 demonstrated a trend in reducing the FTK latency (RSWSRT; Median = 4.0 vs. 4.0; Q1 = 2.0 vs. 2.0; Q3 = 4.5 vs. 5.5, *p* = 0.083). There was no significant difference between W1 vs. W2 (RSWSRT, *p* = 0.317).

Red light exposure for a 2 week period again had little or no effect upon the ETF latency (SFAR, Median for PRE = 3.0; W1 = 3.0; W2 = 2.0: Q1 for PRE = 1.0; W1 = 1.0; W2 = 1.5: Q3 for PRE = 4.5 W1 = 4.5; W2 = 4.5, *p* = 0.472). Withdrawing light from patients showed a significant worsening over the 2 week period of observation. (SFAR, Median for PRE = 3.0; W1 = 3.0; W2 = 3.0: Q1 for PRE = 2.0; W1 = 2.0; W2 = 2.5: Q3 for PRE = 4.0; W1 = 4.0; W2 = 4.5, *p* = 0.039). While the difference between PRE and W1 in this comparison was not significantly different (RSWSRT, *p* = 0.317) the difference between PRE and W2 was (RSWSRT, *p* = 0.046) and the difference between W1 and W2 showed a weak trend (Wilcoxon Signed Rank Test, *p* = 0.83). With the median scores being equal across 3 time groups in the polychromatic treated group and the NLG, the direction of the change as revealed by the interquartile range illustrates a very slight deterioration from PRE to W2, although it may be concluded that this was more severe in the NLG.

Similarly, the clinical score for tremor on the left tremor score varied only slightly across the three treatment groups but was most evident in the NLG. Daily exposure to polychromatic light did not change the global tremor score for this parameter (SFAR, Median PRE = Median for PRE = 2.0; W1 = 2.0; W2 = 2.0: Q1 for PRE = 0.0; W1 = 0.0; W2 = 0.0: Q3 for PRE = 3.0; W1 = 3.0; W2 = 3.5, *p* = 0.472.

In addition, red light exposure for a 2 week period again had little or no effect upon the ETF latency (SFAR, Median for PRE = 2.0; W1 = 2.0; W2 = 3.0: Q1 for PRE = 0.5; W1 = 0.5; W2 = 0.5: Q3 for PRE = 3.5; W1 = 3.0, W2 = 3.0, *p* = 0.368). Withdrawing light from patients showed a significant worsening over the 2 week period of observation. (SFAR, Median for PRE = 2.0; W1 = 2.0; W2 = 2.0: Q1 for PRE = 0.5; W1 = 0.5; W2 = 0.5: Q3 for PRE = 3.0; W1 = 3.0; W2 = 3.5, *p* = 0.050). While the difference between PRE and W1 in this comparison was not significantly different (RSWSRT, *p* = 0.317), the difference between PRE and W2 showed a weak trend (RSWSRT, *p* = 0.083) and the difference between W1 and W2 also showed a weak trend (RSWSRT, *p* = 0.83). Equal median scores across the 3 times of assessment in the NLG again required examination of the interquartile range illustrates that the brunt of deterioration on this parameter was on W2.

The tremor score for the NLG for both left and right sides combined also showed a significant worsening when light exposure was discontinued. Daily exposure to polychromatic light did not change the global tremor score for this parameter (SFAR, Median PRE = Median for PRE = 1.0; W1 = 1.0; W2 = 1.0: Q1 for PRE = 0.0; W1 = 0.0; W2 = 0.0: Q3 for PRE = 2.5; W1 = 2.0; W2 = 2.0, *p* = 0.670). Similarly, red light exposure for a 2 week period again had little or no effect upon the ETF latency (SFAR, Median for PRE = 2.0; W1 = 2.0; W2 = 2.5: Q1 for PRE = 0.0; W1 = 0.0; W2 = 0.0: Q3 for PRE = 25; W1 = 3.0; W2 = 3.0, *p* = 0.417). Withdrawing light from patients showed a significant worsening over the 2 week period of observation. (SFAR, Median for PRE = 2.0; W1 = 2.0; W2 = 2.0: Q1 for PRE = 0.0; W1 = 0.0; W2 = 0.0: Q3 for PRE = 3.0; W1 = 3.0; W2 = 3.0, *p* = 0.417). While the difference between PRE and W1 in this comparison was not significantly different (RSWSRT, *p* = 0.317), the difference between PRE and W2 showed a strong trend (Wilcoxon Signed Rank Test, *p* = 0.059) while the difference between W1 and W2 was significant (RSWSRT, *p* = 0.046). Examination of the interquartile range did not clarify the direction of change however the high score values did for this group with increasing high scores occurring from PRE to W2 in small increments (PRE = 3.0; W1 = 4.0; W2 = 5.0).

Changes were seen in the scores on the Fatigue section of the BBS for the patients continued on polychromatic light and there was a reduction in this score which was revealed as a weak trend as the main effect (SFAR, *p* = 0.097). Further comparison between PRE and W1 for this group revealed no significant effect (Median = 1.0 vs. 1.0; Q1 = 0.0 vs. 0.0; Q3 = 4.0 vs. 4.5, RSWSRT, *p* = 0.317), while the comparison between PRE and W2 demonstrated a trend (Median = 1.0; Q1 = 0.0; Q3 = 0.5, RSWSRT, *p* = 0.083). There was no significant difference between W1 vs. W2 (Medians, Q1 and Q3 values stated above, RSWSRT, *p* = 0.157).

Red light exposure for a 2 week period revealed no significant change on the BBS fatigue scale (SFAR, Median for PRE = 3.0; W1 = 3.0; W2 = 3.0: Q1 for PRE = 2.0; W1 = 0.0; W2 = 0.5: Q3 for PRE = 3.0; W1 = 3.0; W2 = 9.5, *p* = 0.011). Withdrawing light from patients showed a significant worsening over the 2 week period of observation (SFAR, Median for PRE = 3.0; W1 = 3.0; W2 = 3.0: Q1 for PRE = 2.0; W1 = 2.0; W2 = 2.0: Q3 for PRE = 4.0; W1 = 6.0; W2 = 7.0, *p* = 0.039). While the difference between PRE and W1 in this comparison showed a strong trend toward worsening (RSWSRT, *p* = 0.059) the difference between PRE and W2 was significant (RSWSRT, *p* = 0.041) and the difference between W1 and W2 was significant as well (RSWSRT, *p* = 0.046). With the median scores being equal the direction of the change was revealed by examining the interquartile ranges revealing deterioration from PRE to W2, permitting the conclusion that fatigue worsened when light treatment was withdrawn.

The number of awakenings (BBS Scale) for the patients continued on polychromatic light also changed as there was a reduction in this score which was not significant (SFAR, *p* = 0.209). However, while comparison between PRE and W1 for this group revealed no significant difference, (Median = 2.0 vs. 2.0; Q1 = 2.0 vs. 1.25; Q3 = 4.0 vs. 4.0, RSWSRT, *p* = 0.197), comparison between PRE and W2 demonstrated a significant difference (Median = 2.0 vs. 2.0; Q1 = 2.0 vs. 1.0; Q3 = 4.0 vs. 2.5, RSWSRT, *p* = 0.043). There was no significant difference between W1 vs. W2 (Medians, Q1 and Q3 values stated above, RSWSRT, *p* = 0.234).

Red light exposure for a 2 week period again had little or no effect upon the EFT latency (SFAR, Median for PRE = 2.0; W1 = 2.0; W2 = 2.0: Q1 for PRE = 1.0; W1 = 1.0; W2 = 1.25: Q3 for PRE = 2.75; W1 = 2.5; W2 = 2.5, *p* = 0.939). Similarly, withdrawing light from patients was without significant effect (SFAR, Median for PRE = 1.5; W1 = 2.0; W2 = 2.0: Q1 for PRE = 1.25; W1 = 1.5; W2 = 1.0: Q3 for PRE = 2.25; W1 = 3.0; W2 = 2.75, RSWSRT, *p* = 0.840).

There were some instances where continued polychromatic, red light or withdrawal of polychromatic light on variables examined demonstrated little or no effect. Weak trends were sometimes seen on the MDSUPDRS score in the RLG and NLG treatment groups where slight worsening was observed. In addition, a worsening in the bradylogia score on the BBS was also occasionally observed for these groups but none were statistically significant.

## Discussion

The present results demonstrate that continued exposure to polychromatic light over a 2 week period results in incremental improvement in motor and psychiatric parameters associated with PD. In all studies to date the utility of BT in this disorder has been determined on the basis of studies that have administered light to *de novo* patents (7, 11). However, with our experience with the application of BT (6, 8), we have identified numerous technical problems in the therapeutic application of light that frequently interferes with efficacy and which have to be overcome before the patient can experience optimal therapeutic benefit. Such technical problems include positioning of the light source, compliance, pre-existing ocular disease, light sensitivity, consistency in time of administration, polypharmacy, DA replacement overdosing, sleep hygiene and light induced narcolepsy, to name only a few. Such problems are typically resolved in routine neurological practice over the first few visits to the clinic and to minimize their interference during the study, patients were admitted into the study with at least 4 months prior exposure to BT. The obvious benefit of implementing such a paradigm is that it eliminates many of the intervening variables that routinely interfere with effective treatment thereby reducing the “noise” in the sample.

Upon closer examination of previous studies implementing BT in the treatment of PD steps need to be taken to determine the best approach for refining the development of BT for use in this disorder. For example, in the same way that pharmacokinetics and pharmacodynamics are performed for any drug that undergoes development for clinical use, photokinetics and photodynamics should also be performed to determine the best use of light in any clinical application. Even though the use of BT for SAD has been implemented since 1984 (1), there is, as of yet, no established standard regulating the optimal therapeutic transmission for the treatment of any disorder, including PD (6–9, 11), and this problem has been acknowledged (26). In fact, current studies implementing BT for PD select commercial light sources on the basis of emission from the source at a given distance (6–8, 27) but, to the best of our knowledge, no one has yet determined how this translates to the amount of light that actually hits the retina. This may explain the variation of therapeutic effects seen in the present study compared to the therapeutic effects reported in controlled studies implementing BT in PD (7–9, 11). Comparison of the photodynamics of different devices and the careful, consistent positioning of each device can serve to optimize the therapeutic effect. This is complicated further by the complexity of the visual system and the juxtaposition of its subsystems as well as the presence of eye disease, which can vary from patient to patient. Studies examining critical light source positioning, compliance and light frequency are the subjects of our current research.

The mechanism of action underlying the therapeutic effect of BT is yet to be addressed. Recent studies have proposed that retinal system controlling motor function participate in the PD inducing and therapeutic effects seen after intravitreal injections of toxins and anti-PD drugs, respectively (28, 29). One mechanism behind the therapeutic effect of BT that has been recently resurrected (11) suggests that light causes release of retinal DA (27). However, there are findings that are contrary to this interpretation. For example, any direct anatomical or humeral mechanism that could link increased retinal DA with the facilitation of NSD function is unknown. It is intriguing that the discovery describing retinal DA deficiency in PD patients did not occur until almost two decades after the discovery of NSD deficiency, leaving the link between the functional significance of their relationship unrevealed. On this basis, interpretations suggesting that therapeutic effects achieved by increasing retinal DA release with BT are unjustifiable (27). Secondly, larger open label studies have utilized BT to improve the primary and secondary symptoms of PD. In doing so this approach has reduced the incidence of dyskinaesia resulting from DA replacement (9). Involuntary movement in PD is associated with long term, increasing doses of DA replacement and one might well predict that if increasing retinal DA was the mechanism by which BT works then increased dyskinaesia would occur but this is not reported (8, 9). Clearly, the mechanisms by which BT might be rendering its therapeutic effects is an important area of research yet to be fully explored.

The effect of the different treatments on overall MDSUPDRS score illustrate that patients maintained on polychromatic light show significant improvement of up to 16 points during the 2 week period of observation not seen in the RLG or NLG. Analysis of the subsections showed further that the improvement was primarily in sections I and II being mentation, behavior and mood and activities of daily living, respectively. While there was a 60% improvement in section I scores after exposure to polychromatic light over the 2 week period of observation, this was obvious at 1 week after commencing the study and was maintained for the duration. Similarly, but at a magnitude of improvement being half of the PLG, was the improvement in this score on Part I for the RLG group seen with no change in the group for which light was discontinued. This suggests to us that the effect is dose dependent with the number of photons affecting retinal photoreceptors being significantly less than in the RLG compared to the PLG (30). Alternatively, this might be related to the reparative effect of near infrared light purported to occur in PD and other neurological disorders (31). However, given that the red light exposure used in the present study also increased the severity of fatigue, as revealed by the ESS scores, we maintain a conservative interpretation of this finding. Both the magnitude of this effect and the lack of effect of red light on any other parameter would suggest that this is a fortuitous finding. However, future studies employing a larger cohort may reveal the importance of this finding. A similar improvement in daily activities (Part II) was seen in the PLG while the RLG and NLG showed no significant change. At first glance this would suggest that motor parameters were not affected by light administration but previous research has shown that the changes in motor function are very incremental after exposure to light as compared to the expected response to DA replacement (8). This suggests to us that motor function would not be expected to change dramatically within the short time period implemented in the present study. Additional confirmation of the slower rate of recovery of motor parameters is confirmed by results on the timed motor tests whereby EFT and FTK all improved in the PLG while bradykinaesia and tremor deteriorated in the group where light was withdrawn. This represents an improvement in motor function as depicted in timed motor tests ranging from 0.05 to 1.2 s per 2 weeks while deterioration in the group where light was discontinued developed at a similar rate. This, combined with the possibility that the MDSUPDRS may not be adequately sensitive to pick up the subtle changes in motor function that occur on a day to day basis, could account for the lack of observable change in sections 2 and 3 of the MDSUPDRS. This is supported further by results from the Mobility Section Score of the PDQ 39 whereby an 80% improvement was observed between the pre score and W2 only in the PLG. Previous research (6, 8) has also intimated that improvement in non-motor symptoms occurs more rapidly than with motor function and this is confirmed by the present results.

Continued light administration was very effective in improving psychological parameters. For example, the discomfort, stigma, and communication scales on the PDQ-39 all improved between 50 and 100% of pre-test values while the RLG and NLG remained unchanged. There was a significant improvement in the PDQ-39 emotion scale for both the NLG and the PLG although the magnitude of change in the NLG was much less than that exhibited in the PLG. The response in the NLG might well have been due to the relief experienced with the act of not being tied to the treatment regimen that patients often lament during the course of lengthy BT, which we have encountered in the clinic over the past 21 years (8). Given the short duration of observation in the present study, our data favor this interpretation given that we routinely have observed slow deterioration when light is discontinued. The BDI-II and BAI demonstrate further that psychological parameters are noticeably affected by ongoing BT. For example, scores on the BDI improved between 25 to 50% during the course of the trial, even though all patients were maintained on BT prior to commencing the trial. This may also account for the relatively low score that all groups exhibited during the study, and demonstrates that exposure to polychromatic light continues to exert an antidepressant effect for several years after treatment commences (8). Similarly, improvement in the BAI score in the PLG ranging from 50% to almost 70% during the course of treatment also demonstrates the potential of this treatment as an anxiolytic that would be beneficial in a disease plagued with side effects such as anxiety and depression (12, 32, 33). To improve these primary, non-motor symptoms in a disease that is complicated by polypharmacy, implementation of a non-invasive technique would contribute to an improved quality of life in these patients.

Sleep related parameters also showed significant improvement during the course of the 2 week trial. While the scores on the ESS during the pre-test control session were low this may well reflect the efficacy of light on this parameter as patients were maintained for at least 4 months prior to commencing the trial. Those patients continuing with polychromatic light showed a score reduction of about 50% over the 2 week duration of the trial while analysis of the group receiving red light showed a significant increase in sleepiness as intimated by more detailed analysis revealed by examining the Q3 values. In addition the results from the BBS scale regarding assessment of number of awakenings and fatigue, while the number of awakenings decreased in the PLG, those treated with red light or were BT withdrawn showed no significant changes. Global Clinical assessment of patient fatigue as depicted on the BBS scale showed that the NLG displayed an increasing severity of fatigue during the 2 weeks of treatment. These results corroborate previous claims that polychromatic light exposure at strategic times is beneficial in treating various aspects of sleep (11, 34, 35) which commonly occur in PD (6, 8). Bearing in mind that the population of PD patients treated in the present study had already shown improvement in sleep parameters prior to commencing the study, further studies examining the effects of BT in *de novo* patients might well demonstrate a more dramatic effect than the present results.

The time of light administration during the L/D cycle is also an important factor to consider when implementing BT. Previous work in SAD (36) and the first controlled studies in PD (7, 11) all implemented morning light exposure in PD patients. However, previous case studies and longitudinal studies (6, 8) applied light in the evening, just prior to the onset of the dark phase which was justified on the basis of work demonstrating that medicated PD patients were phase advanced (13, 15). Continuation of this paradigm in the present study may account for the more robust effect on the various parameters in the MDSUPDRS, PDQ-39, the BDI-II and the BAI compared to other controlled studies (7, 11). However, other major differences between the present study and previous work also exist, such as the use of BT in BT-experienced vs. *de novo* patients and this too might account for the more robust effect. Whichever the case might be, this illustrates that the use of BT in PD is yet to be refined to obtain the optimal therapeutic effect and caution should be exerted in drawing conclusions as to the optimal paradigm at this early stage.

Recent studies implementing light therapy in PD (11, 27) have confirmed our suggestion as to the possible role of retinal DA in the therapeutic effect of BT (4, 28). This concept arises from older studies demonstrating that the retina contains melanocytes that respond to light and dark (37) and that these cells are also present in the NSD and pineal (4, 38). Their interrelationship has been hypothesized to mediate the effects of toxins in causing PD (29) and in the therapeutic effect of DA replacement (28, 39). In fact, recent studies have defined neurochemical systems in the retina that exert control over deep brain systems controlling movement (40) and embrace circadian involvement (41). However, caution should be exercised in ascribing a therapeutic response solely to retinal DA function given that we have never observed enhanced dyskinaesia and other adverse effects with extended use of light in PD patients experiencing DA overdosing phenomena (6, 8, 22). Ongoing research examining the interplay of the NSD and circadian systems in PD will serve to elucidate the role of each system in the onset and progression of the disease and will enable the development of improved treatment strategies.

One possible limitation of the present study was that we did not control for whether patients were “ON” or “OFF” during each stage of formal assessment. In one regard, this was not of major concern as a result of two factors. First of all, daily circadian charts were completed during each assessment with fluctuations in treatment response recorded on a 24 h basis. OFF periods occurred rarely in our patient population during the assessments and most patients were scheduled at approximately the same time of day for each of the 3 assessments. Given that OFF periods were also assessed in the MDSUPDRS, and that we compared performance during the PRE session with that of W1 and W2, this parameter could also be monitored as a dependent variable sensitive to the beneficial effects of BT.

The most obvious limitation of this study is the small numbers employed. This is best exemplified in the mean scores on the 5 assessments employed in the study with the lowest baseline scores occurring during the PRE measurement. While the three groups were not significantly different during this measurement the critical analysis for the present study was change in score, for each group, during the 2 week test period. Only the PLG continued to improve reliably during the course of the study. Future studies with a larger cohort observed for a longer period of treatment will help to elucidate the strength of the effect observed in the present study.

In conclusion, the present results consistently demonstrate that continued exposure to polychromatic light, just prior to commencement of the dark phase, provides symptomatic improvement in primary motor and non-motor symptoms of PD. While the present study is small there is considerable internal consistency in the majority of therapeutic effects that can be attributed to the continued BT at strategic times. There are controlled studies (7, 11) and epidemiological studies demonstrating that light exposure at night can reduce the incidence of PD (42), or that geographical locations exist where reduced ambient light is associated with an increase in the incidence of this disorder (43, 44). Such data support the possibility that light might well be valuable in symptomatic treatment and improving the quality of life in PD patients. In addition, LT might also serve as an adjunct treatment to reduce or eliminate the invasive effects of DA replacement (8) or surgical techniques (22, 45, 46) used routinely in the treatment of PD where the side effects often become worse than the disease itself (22). The present results also provide further support for the recent suggestion that the circadian system is involved in PD (6–8, 11, 35). While the mechanism by which this is achieved remains somewhat elusive, we hypothesize that the retina may play a major role in defining how the circadian system is involved (28, 40, 41, 47) and from this will emerge less invasive therapeutic approaches. Whatever the outcome of such exploration, it is becoming increasingly evident that very subtle changes in neurochemistry at targets distant from the NSD can evoke a significant therapeutic response in PD.

## Author contributions

GW and CF contributed to the conception and design of the study. CF and JB served as monitors and organized the database while all authors undertook analysis. All authors also participated in manuscript preparation, revision, and approval of the final version. The corresponding author takes responsibility for the submission and review of the publication process. The corresponding author also takes responsibility ensuring the accuracy of authorship determination, ethics approval clinical trial registration and conflict of interest submissions as well as for any publication issues.

### Conflict of interest statement

The authors declare that the research was conducted in the absence of any commercial or financial relationships that could be construed as a potential conflict of interest.

## References

[1] Rosenthal NE Sack DA Gillin JC Lewy AJ Goodwin FK Davenport Y. Seasonal affective disorder. A description of the syndrome and preliminary findings with light therapy. Arch Gen Psychiatry (1984) 41:72–80. 10.1001/archpsyc.1984.01790120076010 6581756

[2] Nussbaumer B Kaminski-Hartenthaler A Forneris CA Morgan LC Sonis JH Gaynes BN Light therapy for preventing seasonal affective disorder. Cochrane Database Syst Rev. (2015) 8:CD011269 10.1002/14651858.CD011269.pub2 26558494

[3] Wirz-Justice A Krauchi K Brunner DP Graw P Haug HJ Leonhardt G. Circadian rhythms and sleep regulation in seasonal affective disorder. Acta Neuropsychiatr. (1995) 7:41–3. 10.1017/s0924270800037522 26965348

[4] Willis GL. Parkinson's disease as a neuroendocrine disorder of circadian function: dopamine-melatonin imbalance and the visual system in the genesis and progression of the degenerative process. Rev Neurosci. (2008) 19:245–316. 10.1515/REVNEURO.2008.19.4-5.245 19145986

[5] McEnany GW Lee KA. Effects of light therapy on sleep, mood, and temperature in women with nonseasonal major depression. Issues Ment Health Nurs. (2005) 26:781–94. 10.1080/01612840591008410 16126652

[6] Willis GL Turner EJ. Primary and secondary features of Parkinson's disease improve with strategic exposure to bright light: a case series study. Chronobiol Int. (2007) 24:521–37. 10.1080/07420520701420717 17612949

[7] Paus S Schmitz-Hubsch T Wullner U Vogel A Klockgether T Abele M. Bright light therapy in Parkinson's disease: a pilot study. Mov Disord. (2007) 22:1495–8. 10.1002/mds.21542 17516492

[8] Willis GL Moore C Armstrong SM. A historical justification for and retrospective analysis of the systematic application of light therapy in Parkinson's disease. Rev Neurosci. (2012) 23:199–226. 10.1515/revneuro-2011-0072 22499678

[9] Artemenko AR Levin I. [The phototherapy of parkinsonism patients]. Zh Nevrol Psikhiatr Im SS Korsakova (1996) 96:63–6. 8992840

[10] Videnovic A Noble C Reid KJ Peng J Turek FW Marconi A. Circadian melatonin rhythm and excessive daytime sleepiness in Parkinson disease. JAMA Neurol. (2014) 71:463–9. 10.1001/jamaneurol.2013.6239 24566763 PMC4078989

[11] Videnovic A Klerman EB Wang W Marconi A Kuhta T Zee PC. Timed light therapy for sleep and daytime sleepiness associated with parkinson disease: a randomized clinical trial. JAMA Neurol. (2017) 74, 411–418. 10.1001/jamaneurol.2016.5192 28241159 PMC5470356

[12] Rutten S Vriend C van den Heuvel OA Smit JH Berendse HW van der Werf YD. Bright light therapy in Parkinson's disease: an overview of the background and evidence. Parkinson's Dis. (2012) 2012:767105. 10.1155/2012/767105 23320250 PMC3540893

[13] Bordet R Devos D Brique S Touitou Y Guieu JD Libersa C. Study of circadian melatonin secretion pattern at different stages of Parkinson's disease. Clin Neuropharmacol. (2003) 26:65–72. 10.1097/00002826-200303000-00005 12671525

[14] Fertl E Auff E Doppelbauer A Waldhauser F. Circadian secretion pattern of melatonin in Parkinson's disease. J Neural Transm Parkinson's Dis Dement Sec. (1991) 3:41–7. 10.1007/BF02251135 2064730

[15] Fertl E Auff E Doppelbauer A Waldhauser F. Circadian secretion pattern of melatonin in de novo parkinsonian patients: evidence for phase-shifting properties of l-dopa. J Neural Transm Parkinson's Dis Dement Sec. (1993) 5:227–34. 10.1007/BF02257677 8369102

[16] Catala MD Canete-Nicolas C Iradi A Tarazona PJ Tormos JM Pascual-Leone A. Melatonin levels in Parkinson's disease: drug therapy versus electrical stimulation of the internal globus pallidus. Exp Gerontol. (1997) 32:553–8. 10.1016/S0531-5565(96)00173-8 9315456

[17] Ralph CL Correlations of melatonin content in pineal gland, blood, and brain of some birds and mammals. Am Zool. (1976) 16:35–43. 10.1093/icb/16.1.35

[18] Roth WD Wurtman RJ Altschule MD Morphologic changes in the pineal parenchyma cells of rats exposed to continuous light or darkness. Endocrinology (1962) 71:888–92. 10.1210/endo-71-6-888 13975335

[19] Kisaalita NR Roditi D Robinson ME. Factors affecting placebo acceptability: deception, outcome, and disease severity. J Pain (2011) 12:920–8. 10.1016/j.jpain.2011.02.353 21816353 PMC3150516

[20] Colloca L Miller FG. Role of expectations in health. Curr Opin Psychiatry (2011) 24:149–55. 10.1097/YCO.0b013e328343803b 21248640

[21] van der Graaf R van Delden JJ Equipoise should be amended, not abandoned. Clin Trials (2011) 8:408–16. 10.1177/1740774511409600 21746767

[22] Willis GL Moore C Armstrong SM. Breaking away from dopamine deficiency: an essential new direction for Parkinson's disease. Rev Neurosci. (2012) 23:403–28. 10.1515/revneuro-2012-0037 23089606

[23] Gagne AM Levesque F Gagne P Hebert M. Impact of blue vs red light on retinal response of patients with seasonal affective disorder and healthy controls. Prog Neuro Psychopharmacol Biol Psychiatry (2011) 35:227–31. 10.1016/j.pnpbp.2010.11.009 21094670

[24] Reme CE Rol P Grothmann K Kaase H Terman M. Bright light therapy in focus: lamp emission spectra and ocular safety. Technol Health Care (1996) 4:403–13. 9042691

[25] Duvoisin RC Golbe LI Lepore FE. Progressive supranuclear palsy. Can J Neurol Sci Le J Can Des Sci Neurol. (1987) 14(3 Suppl.):547–54. 3315157

[26] Aarts MPJ Rosemann ALP. Towards a uniform specificationof light therapydevices for the treatment of affective disorders and use for non-image forming effects: radiant flux. J Affect Disord. (2018) 235:142–9. 10.1016/j/jad2018.04.020. 29656258

[27] Videnovic A Klerman EB Zee PC. Light therapy promoting dopamine release by stimulating retina in parkinson disease-reply. JAMA Neurol. (2017) 74:1268–9. 10.1001/jamaneurol.2017.1909 28806434

[28] Willis GL. Intraocular microinjections repair experimental Parkinson's disease. Brain Res. (2008) 1217:119–31. 10.1016/j.brainres.2008.03.083 18502399

[29] Willis GL Moore C Armstrong SM. Parkinson's disease, lights and melanocytes: looking beyond the retina. Sci Rep. (2014) 4:3921. 10.1038/srep03921 24473093 PMC5379242

[30] Revell VL Arendt J Terman M Skene DJ. Short-wavelength sensitivity of the human circadian system to phase-advancing light. J Biol Rhythms (2005) 20:270–2. 10.1177/0748730405275655 15851533

[31] Hamblin MR. Shining light on the head: photobiomodulation for brain disorders. BBA Clin. (2016) 6:113–24. 10.1016/j.bbacli.2016.09.002 27752476 PMC5066074

[32] Prasuhn J Piskol L Vollstedt EJ Graf J Schmidt A Tadic V. Non-motor symptoms and quality of life in subjects with mild parkinsonian signs. Acta Neurol Scand. (2017) 136:495–500. 10.1111/ane.12760 28345787

[33] Khan MA Quadri SA Tohid H. A comprehensive overview of the neuropsychiatry of Parkinson's disease: a review. Bull Menninger Clin. (2017) 81:53–105. 10.1521/bumc.2017.81.1.53 28271905

[34] Wirz-Justice A. Temporal organization as a therapeutic target. Dialogues Clin Neurosci. (2012) 14:335–7. 23393412 10.31887/DCNS.2012.14.4/awjustice PMC3553567

[35] Videnovic A. Management of sleep disorders in Parkinson's disease and multiple system atrophy. Mov Disord. (2017) 32:659–668. 10.1002/mds.26918 28116784 PMC5435534

[36] Wirz-Justice A Benedetti F Terman M Chronotherapeutics for Affective Disorders. Basel: S. Karger A. G. (2009).

[37] Dubocovich ML. Melatonin is a potent modulator of dopamine release in the retina. Nature (1983) 306:782–4. 10.1038/306782a0 6656879

[38] Barbeau A. Manganese and extrapyramidal disorders (a critical review and tribute to Dr. George C. Cotzias). Neurotoxicology (1984) 5:13–35. 6538948

[39] Willis GL. The therapeutic effects of dopamine replacement therapy and its psychiatric side effects are mediated by pineal function. Behav Brain Res. (2005) 160:148–60. 10.1016/j.bbr.2004.11.030 15836910

[40] Willis GL Freelance CB. Neurochemical systems of the retina involved in the control of movement. Front Neurol. (2017) 8:324. 10.3389/fneur.2017.00324 28725212 PMC5497141

[41] Willis GL Freelance CB. Emerging preclinical interest concerning the role of circadian function in Parkinson's disease. Brain Res. (2017) 1678:203–13. 10.1016/j.brainres.2017.09.027 28958865

[42] Chen H Schernhammer E Schwarzschild MA Ascherio A. A prospective study of night shift work, sleep duration, and risk of Parkinson's disease. Am J Epidemiol. (2006) 163:726–30. 10.1093/aje/kwj096 16495472

[43] de Pedro Cuesta J. Studies on the prevalence of paralysis agitans by tracer methodology. Acta Neurol Scand Suppl. (1987) 112:1–106. 3303809

[44] de Pedro-Cuesta J Stawiarz L. Parkinson's disease incidence: magnitude, comparability, time trends. Acta Neurol Scand. (1991) 84:382–8. 1776385 10.1111/j.1600-0404.1991.tb04974.x

[45] Hu K Moses ZB Hutter MM Williams Z. Short-term adverse outcomes after deep brain stimulation treatment in patients with Parkinson disease. World Neurosurg. (2017) 98:365–74. 10.1016/j.wneu.2016.10.138 27826085

[46] Fang JY Tolleson C. The role of deep brain stimulation in Parkinson's disease: an overview and update on new developments. Neuropsychiatr Dis Treat. (2017) 13:723–32. 10.2147/ndt.s113998 28331322 PMC5349504

[47] Willis GL Freelance CB. The effect of directed photic stimulation of the pineal on experimental Parkinson's disease. Physiol Behav. (2017) 182:1–9. 10.1016/j.physbeh.2017.09.014 28919247

